# Mainstream reintegration of COVID-19 survivors and its implications for mental health care in Africa

**DOI:** 10.11604/pamj.2020.36.366.25115

**Published:** 2020-08-28

**Authors:** Edward Kwabena Ameyaw, John Elvis Hagan, Bright Opoku Ahinkorah, Abdul-Aziz Seidu, Thomas Schack

**Affiliations:** 1 The Australian Centre for Public and Population Health Research, Faculty of Health, University of Technology Sydney, Sydney, Australia,; 2 Department of Health, Physical Education and Recreation, University of Cape Coast, Cape Coast, Ghana,; 3 Neurocognition and Action-Biomechanics-Research Group, Faculty of Psychology and Sport Sciences, Bielefeld University, Bielefeld, Germany,; 4 Department of Population and Health, University of Cape Coast, Cape Coast, Ghana,; 5 College of Public Health, Medical and Veterinary Sciences, James Cook University, Townsville, Queensland 4811, Australia

**Keywords:** COVID-19, infectious disease, pandemic, mental health, depression, emotional distress, psychosocial distress

## Abstract

The novel coronavirus pandemic has undoubtedly emerged as a serious public health threat in many societies across the world. Due to the sporadic and unpredictable nature of the pandemic, it is important to admit that the virus can cause psychological distress and emotional instability that might impact on people in diverse ways at the individual, community and national levels, with serious mental health implications (e.g. depression, mood disorders, obsessive-compulsive disorder, other anxiety disorders). Due to the weak healthcare challenges inherent in Africa, these mental health challenges require urgent redress to ensure mental health well-being for all, especially COVID-19-positive patients who have recovered (i.e. survivors). This essay outlines some of these challenges and offers strategies to address them. Broader mental health training for facility and community-based health workers are urgently required and should be coordinated within countries with specific guidelines for psychosocial support during outbreaks such as the current pandemic. A framework that promotes reintegration for COVID-19 survivors could also be designed based on context-specific needs through individualized protocols such as the “RAPID-Psychological First Aid [PFA]”. This tool kit, if effectively employed, would help facilitate optimal well-being of the people devoid of any psychological challenges created by the pandemic.

## Essay

Although Africa was lately hit by the novel coronavirus 2019 (COVID-19), the pace of spread in the last few months has been exponential. By 7:00 GMT, 30^th^ June 2020, Africa had 508,114 confirmed cases, with 245,033 recoveries and 11,978 deaths [1]. These statistics clearly indicate that most COVID-19 infected persons in Africa are recovering. The global community have commenced measures to restore routine life activities and economies to embrace the “new normalcy” following numerous instituted measures to halt and reduce the spread of the novel COVID-19 pandemic. Most countries have adopted a phased approach of easing the stringent measures imposed. Some of the specific strategies include increased use and acceptance of electronic approaches (e.g. digital platforms-Zoom conference meetings, e-transactions) as strategies for reducing physical contact and halting the spread of the virus. For individuals recovering from the virus, the effect of COVID-19 on their mental health in Africa could be enormous, given the fragile healthcare systems [2, 3]. Learning from the experience with Ebola, studies have shown that majority of Ebola survivors went through traumatic psychosocial consequences such as depression, anxiety and grief due to loss of loved ones and stigma due to the adverse psychological experiences they had to go through during infection, treatment and post-discharge [4].

Parallel to the Ebola epidemic of 2014-2016, COVID-19 is likely to cause anxiety, depression and post-traumatic stress disorders [3]. With the upsurge of COVID-19 pandemic on the African continent, social stigma and xenophobia have become entrenched in current public discourse and the stark reality [5]. According Kaufman and partners, there have been reported increases in resentment towards COVID-19 positive patients in areas with high infection rates, leading to reports of social stigma by patients with related increased anxiety and depression. Considering the high COVID-19 recovery rate in Africa, a critical dimension that warrants policy and governments´ attention is the approaches to ensure that all persons who recover from the virus are fully restored in all spheres of life (i.e. socially, psychologically, economically, spiritually etc.). Therefore, considerable attempts to control the disease transmission should be context-specific. The low digital literacy, low smartphone penetration and limited internet connection, especially in the rural populations could be a major challenge for service delivery during the period.

Having a well-articulated and comprehensive strategy on how to absorb recovered persons into the African society is invaluable because unlike the western world, diseases are usually attributed to indigenous (e.g. metaphysical) causes in Africa. For instance, more often than not, disease outbreaks are interpreted as punishment for wrongdoing or not treating one´s ancestors well, or perhaps attack by evil or bad spirits [6, 7]. These thinking patterns have traditional and cultural reinforcements that may foster alienation and stigmatisation toward persons who have recovered from COVID-19. For example, maintaining social and cultural resilience factors and coping mechanisms is critical in the African context. The proceeding paragraphs in this essay present the concept of illness behaviour, psychological impact COVID-19 might pose and some suggested measures that may expedite reintegration of recovered persons into mainstream society.

**Conceptualizing COVID-19 illness behavior:** the conceptualization of illness has longstanding history whereby the sick are segregated from healthy individuals [7]. The concept of illness behaviour describes how people respond to bodily indications and the conditions under which they view them as abnormal. Thus, illness behaviour involves how individuals monitor their bodies and interpret their symptoms, take remedial action, and utilize sources of help as well as the more formal healthcare system. Individuals monitor and respond to symptoms and symptom change over the course of an illness and how this affects subsequent behaviour, remedial actions taken, and response to treatment [8]. Hence, responses to COVID-19 illnesses will be mapped by the unpredictability and perceived contagion [9]. According to Jones [10], some chronological accounts of how responses to epidemics manifest include perceived lack of recognition of the magnitude of the problem, followed by public responses that are often grounded in moralistic and mechanistic interpretations. An established premise in literature is that illness experience is fundamentally shaped by sociocultural and social-psychological factors regardless of their genetic, physiological or other biological bases. Therefore, diverse perceptions, appraisals and reactions to illness have a significant influence on the extent to which symptoms might inhibit usual life practices, seek suitable care and patients´ cooperation during treatment [8].

**Psychological impact of COVID-19**: research investigations on the psychological effect of previous infectious diseases (e.g. Middle East respiratory syndrome coronavirus [MERS-CoV]; severe acute respiratory syndrome [SARS]) are akin to the current COVID-19 pandemic [11] Psychological problems such as anxiety, depression, panic attacks, or psychotic symptoms were reported among healthcare workers and the general public. For instance, healthcare professionals who were quarantined, worked in SARS units, or had family or friends who contracted SARS, showed more anxiety, depression, frustration, fear, and post-traumatic stress than colleagues who had no such experience [12, 13]. Similarly, COVID-19 related psychological challenges have also been acknowledged in many scientific studies [14, 15]. Several published studies have revealed prevalence of insomnia, depression and anxiety among different target populations such as healthcare workers and the general population during the COVID-19 in several countries (e.g. China, Italy, Iran, Israel, Singapore, Spain, UK, US [3, 16].

The COVID-19 pandemic has impacted negatively on millions of lives across the world and is likely to cause serious mental health problems among persons with no previous mental challenges as well as worsen the condition of individuals with known mental health illness [3]. These conditions are likely to continue as the virus continues to rise in some populations (e.g. Africa) and post pandemic era. Given that COVID-19 cases are speedily increasing in many countries, including the African countries, psychological challenges may have occurred and will continue to influence millions of people on the African continent. Understanding the psychological impact COVID-19 would provide theoretical evidence for unearthing populations at high-risk that require specific psychological interventions. Such scholarly information would also help plan for needed resources and adjust national and governmental policies in the area of mental health [16]. Clinicians´ awareness of these mental challenges, their correlates, and approaches to control them and incorporate the required needs of specific populations like Africa [17] and other preventive methods essential to manage the spread of COVID-19 are critical [18]. Unfortunately, both theoretical and empirical evidence on the impact of COVID-19 on mental health in Africa are sparse. The WHO has cautioned that Africa could witness as many as 44 million COVID-19 cases and up to 190 000 deaths among Africans, subject to the interventions implemented to curb the virus spread [19]. Hence, if these COVID-19 figures are anything to go by, then the effect on the mental health of individuals in the African continent would be enormous, unless socio-cultural resilience and coping mechanisms are well implemented.

**Addressing mental health challenges in Africa context through practical interventions:** the current upsurge of COVID-19 in Africa and potential psychological impact have both clinical and public health implications. With the continent already identified as COVID-19 high-risk population [19], designing effective interventions for psychologically and emotionally distressed persons at both clinical and community levels would be appropriate. Due to the weak healthcare systems, rolling out psychological services and intervention programs across African countries is crucial. Hence, there is an urgent need to prioritize the public mental health during and post COVID-19 pandemic from a context-specific perspective.

For many African people, seeking healthcare services only happen when all other social means and self-help methods have failed or when disease symptoms get worse [3]. According to Frissa and partner, some social means that people rely on for stress management and other personal health challenges include socializing with others, attendance to faith-based and religious events (e.g. church service) as well as praying and reading of scriptures (e.g. from the Bible, Quran). COVID-19 restrictions in Africa have limited access to these social mechanisms. Across many countries in Africa, government agencies (e.g. Ministries of Health and Information) hold frequent press releases on COVID-19 and publicize important information to the public and in close collaboration with local radio and television stations. To boost services and social resources that are limited due to COVID-19 restrictions, media communication could target self-help measures that are likely to reduce stress and/or emotional distress. Other media educational campaigns should highlight common psychological effects of a pandemic and use case experiences of persons undergoing recovery (e.g. Oluwaseun Osowobi on BBC and testimonies of patients in Burkina Faso) to help others cope better, including issues related to stigmatization and discrimination. Religious services and counseling-related discussions on the pandemic could also be broadcasted on several media platforms repeatedly [3].

Mental health services (e.g. lack of personnel, facilities) are limited in many parts of the world, including Africa. For effective and optimal services to be rendered across the continent, COVID-19 pandemic provides an opportunity to revamp the mental health and social care services sector to improve lives during this period through comprehensive and optimal initiatives. Because many people fall within the low-middle wealth quintile in Africa, many countries depend heavily on community health workers for most healthcare delivery services. Hence, proper training in basic aspects of mental healthcare and engaging the services of these personnel (e.g. psychiatrists, clinical psychologists and mental health nurses) would guide the development of inter-sectoral collaborations to protect and support affected individuals through face-to-face counseling, psychosocial screening, and stigma reduction within local communities [20]. Other specific therapeutic strategies such as the provision of toll-free mental health helplines can effectively be utilized to offer in-depth and anonymous counseling on radio and television and other self-help services, especially for individuals in lower socioeconomic strata [21]. Providing free or cheaper online mental health services to the general public would also be useful [22].

**Rehabilitation or recovery for COVID-19 survivors:** redesigning the mental health pathway that mirrors the COVID-19 patient´s journey from home to hospital and back again is critical towards supportive assistance for affected persons´ reintegration. A framework that can easily be adopted to offer valuable mental healthcare for affected COVID-19 survivors is the “Psychological First Aid (PFA)” protocol. The PFA is a tool kit that provides psychosocial support through decisive early intervention during outbreaks like COVID-19 [23]. It is a coping tool designed to alleviate acute distress and also evaluate the necessity for sustained mental healthcare via empathy and reassurance [24]. PFA creates a conduit for concerted services and managing information among COVID-19 affected persons [25]. PFA emergency management can be offered through different models (e.g. PFA for field workers, PFA for first responders, PFA for listen, protect, and connect).

One of the PFA models that could broadly be used to extend behavioural health surge capacity is the “RAPID” tool kit developed by The Johns Hopkin´s Center for Public Health Preparedness. The RAPID-PFA model offers a five-step approach that evaluates the effectiveness of trained providers (i.e. non-mental health trained public health personnel) in real crisis situations like COVID-19 [26, 27]. The five-step procedures include step one; rapport and reflective listening: used during the interaction with a person in crisis, and the objective is to create initial contact and establish rapport through active and reflective listening techniques like paraphrasing with empathy; step two involves assessment that hinges on the evaluation of psychological and basic physical needs; step three focuses on prioritization by emphasizing the importance of triage principles by targeting on the severity of cases that needs emergent care; step four introduces an intervention that aims to alleviate distress and promote functional capacity using cognitive and behavioural interventions (e.g. positive self-talk, relaxation, imagery), and also offer some basic needs; and step five as the final protocol targets disposition and follow-up that provides continuous process until stabilization of the situation through constant support, meeting client needs, and regular monitoring. Essentially, all the protocols aim to calm and orient emotionally overwhelmed survivors, offer practical help, contact, and engage, provide safety and comfort, and gather information on the present concerns and needs [24]. According Birkhead and Vermeulen [24], PFA is a useful tool for clinicians, other analogous personnel and survivors to effectively manage stress-related reactions after traumatic events like the COVID-19 pandemic.

**Conclusions**: COVID-19 will continually unsettle lives across many societies, including Africa. As governments use multifaceted strategies to manage COVID-19, issues related to mental healthcare and psychosocial support should also be given a priority and integrated as part of the overall framework for managing the pandemic, including survivors of the virus. Broader mental health training for facility and community-based health workers are urgently required and should be coordinated within countries with specific guidelines for psychosocial support during outbreaks such as the current pandemic. These mental health services should readily be available and easily accessible across countries. Adopting a proactive strategy that accounts for longer term rather than short-term behavioural responses to the pandemic would be desirable. The use of reintegration approaches for COVID-19 survivors, delivered on the basis of context-specific needs through individualized protocol like the RAPID-PFA that would mastermind optimal well-being of the people devoid of any psychological challenges created by the pandemic might be useful. These strategies should consider cultural characteristics and resources required for social support and resilience of these survivors and the general public.
